# Pulse grazing by reindeer (*Rangifer tarandus*) can increase the phylogenetic diversity of vascular plant communities in the Fennoscandian tundra

**DOI:** 10.1002/ece3.8131

**Published:** 2021-10-07

**Authors:** Kate Gibson, Johan Olofsson, Arne Ø. Mooers, Melanie J. Monroe

**Affiliations:** ^1^ Department of Biology Simon Fraser University Burnaby BC Canada; ^2^ Department of Ecology and Environmental Science Umeå University Umeå Sweden

**Keywords:** biodiversity, community structure, grazing, herbivore, phylogenetic diversity

## Abstract

Herbivore grazing is an important determinant of plant community assemblages. Thus, it is essential to understand its impact to direct conservation efforts in regions where herbivores are managed. While the impacts of reindeer (*Rangifer tarandus*) grazing on plant biodiversity and community composition in the Fennoscandian tundra are well studied, the impact of reindeer grazing on phylogenetic community structure is not. We used data from a multiyear quasi‐experimental study in northern Fennoscandia to analyze the effect of reindeer grazing on plant community diversity including its phylogenetic structure. Our study design used a permanent fence constructed in the 1960s and temporary fences constructed along the permanent fence to expose plant communities to three different grazing regimes: light (almost never grazed), pulse (grazed every other year), and press (chronic grazing for over 40 years). Similar to previous studies on low productivity ecosystems in this region, the species richness and evenness of plant communities with pulse and press grazing did not differ from communities with light grazing. Also consistent with previous studies in this region, we observed a transition from shrub‐dominated communities with light grazing to graminoid‐dominated communities with pulse and press grazing. Interestingly, communities with pulse, but not press, grazing were more phylogenetically dispersed than communities with light grazing. If grazing pulses can increase the phylogenetic diversity of plant communities, our result suggests changes in reindeer management allowing for pulses of grazing to increase phylogenetic diversity of plant communities.

## Graphical abstract

We used data from a multiyear experimental study in northern Fennoscandia to analyze the effect of reindeer (*Rangifer tarandus*) grazing on plant community diversity including its phylogenetic structure. Interestingly, communities with pulse, but not press, grazing were more phylogenetically dispersed than communities with light grazing. Our results have important implications for our understanding of how herbivore grazing drives plant community assembly and for the management of reindeer in Fennoscandia if pulses of grazing can increase the phylogenetic diversity of plant communities.

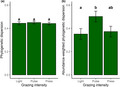

## 1 INTRODUCTION

Herbivore grazing is an important factor influencing plant community assemblages directly through the physical removal of plant species (Lubchenco, 1978) and indirectly by altering patterns of ecosystem productivity (McNaughton et al., 1988) and nutrient availability (Mazumder et al., 1988). As a result, herbivores may alter species composition (Augustine & McNaughton, 1998), measures of richness (Olff & Ritchie, 1998), and the phylogenetic structure (Cavender‐Bares et al., 2009) of plant communities. Plant species richness and evenness (Tilman et al., 1997) and phylogenetic diversity (Faith, 1992) are both important for ecosystem function and productivity (Liu et al., 2018). Thus, understanding the impact of herbivore grazing on these measures is essential for directing conservation efforts in regions where herbivores are managed (Olofsson et al., 2004).

Herbivore grazing can both increase and decrease the diversity of plant species (Olff & Ritchie, 1998; Proulx & Mazumder, 1998). According to the intermediate disturbance hypothesis, plant communities will increase or decrease their biodiversity as a function of the level of disturbance (e.g., grazing) (Connell, 1978; Grime, 1973). Low levels of grazing may increase plant species richness by removing dominant and competitive species and increasing light exposure and nutrient availability (Bakker et al., 2003; Olff & Ritchie, 1998; Proulx & Mazumder, 1998). High levels of grazing, however, may decrease species richness due to insufficient recovery periods, trampling, and erosion (Olff & Ritchie, 1998).

The nutrient availability of an ecosystem may play a role in determining the grazing intensity that results in the maximum biodiversity (Proulx & Mazumder, 1998). Nutrient‐rich ecosystems are expected to experience peak levels of biodiversity at greater grazing intensities because nutrient‐rich ecosystems are usually dominated by a few species. Grazing of the dominant species can prevent their domination and should increase diversity because the plant species are able to quickly respond to disturbance (Huston, 1979; Rosenzweig, 1971). Nutrient‐poor ecosystems, however, are limited by their regrowth ability and thus are expected to achieve the greatest species richness at lower grazing intensities (Proulx & Mazumder, 1998). Thus, high levels of grazing are more likely to result in increased species richness in nutrient‐rich ecosystems but have no effect or result in decreased diversity in nutrient‐poor ecosystems. A positive effect of reindeer grazing in productive sites, and a negative effect in low‐productive sites, has indeed been recorded in a multisite study in the Fennoscandian tundra (Sundqvist et al., 2019).

Herbivore grazing may also alter the phylogenetic structure of communities, though predictions are dependent on the evolutionary history of antiherbivore defense traits that some plant species have evolved, as well as the type of herbivores (Cavender‐Bares et al., 2009). If antiherbivore defense traits are phylogenetically conserved (Loiola et al., 2012; Yessoufou et al., 2013, but see Kursar et al., 2009), then generalist herbivores such as reindeer (Baskin & Danell, 2003) may create a plant community that is more phylogenetically clumped such that communities include more closely related species than expected from the regional pool (Begley‐Miller et al., 2014). However, if antiherbivore defense traits are evolutionarily convergent, a generalist herbivore may increase the phylogenetic dispersion of a community; that is, communities include more distantly related species than expected (Cavender‐Bares et al., 2009). Alternatively, intense competition between distantly related taxa (Mayfield & Levine, 2010) and limited nutrient availability both are expected to drive phylogenetic clumping (Hurteau et al., 2016). If herbivores both (a) decrease competition via removal of dominant plant species from a community and (b) increase nutrient availability via changes in nutrient cycling, herbivore grazing may result in phylogenetic dispersion regardless of the evolutionary history of antiherbivore defense traits. There are, however, no studies addressing the effect of reindeer grazing on phylogenetic diversity.

The most extensive form of human land use in the northern Fennoscandian tundra is grazing by reindeer (*Rangifer tarandus*), and the major populations of reindeer across different regions have increased, decreased, or remained stable over recent decades (Uboni et al., 2016). The effects of reindeer grazing on tundra plant communities are significant (Austrheim & Eriksson, 2001; Bernes et al., 2015; Suominen & Olofsson, 2000; van der Wal, 2006), as demonstrated by the pronounced replacement of dwarf shrubs by graminoids in heavily grazed areas (Olofsson et al., 2001; Sundqvist et al., 2019). The effect of reindeer on plant diversity varies depending on a large number of factors such as the diversity measure used, vegetation types, and climatic conditions (Suominen & Olofsson, 2000; Bernes et al., 2015, see also Scharn et al., 2021). In a Scandinavia‐wide study, Sundqvist et al. (2019) were able to show that at least part of this variation depends on site productivity and grazing intensity: Reindeer grazing decreased species richness in sites with low productivity, but increased species richness in productive sites, and the effects were stronger in sites with higher grazing intensity. Despite a major advance in understanding the effect of reindeer on diversity, we still lack solid knowledge about the effect of different grazing regimes (i.e., continuous press grazing or varying pulse grazing), and no studies have so far addressed the effect of reindeer grazing on phylogenetic community structure, something that may provide insight into the mechanisms driving shifts in community assembly and diversity (Webb et al., 2002). To be able to manipulate reindeer grazing regimes and achieve the pulse and press treatments, we collaborated with reindeer herders.

Here, we study the effects of reindeer grazing on plant community structure using data from a multiyear quasi‐experiment from 2004 to 2007 including varying intensities of grazing: light (almost never grazed), acute (“pulse,” grazed every other year), and chronic (“press,” grazed every year), in the region of Fennoscandia. First, we considered the effect of grazing on the diversity of vascular plant species: Based on the previous work in Fennoscandia, we predicted that in the nutrient‐poor tundra, both pulse and press grazing would have no effect or negative effects on species richness and evenness. Second, we analyzed the effect of grazing on plant species composition, with a prediction that the pulse‐ and press‐grazed areas would see the replacement of dwarf shrubs by graminoids (Olofsson et al., 2001). Finally, we analyzed the effect of grazing on the phylogenetic structure of the vascular plant communities. As reindeer are generalist herbivores (Baskin & Danell, 2003) and antiherbivore traits are generally evolutionarily conserved (Loiola et al., 2012; Yessoufou et al., 2013, but see Kursar et al., 2009), we predicted that grazing would result in appreciable phylogenetic clustering such that species in areas with pulse and press grazing would be more closely related to one another than those in lightly grazed areas.

## 2 MATERIALS AND METHODS

### 2.1 Study site

Our study was conducted in Raisduoddar, a suboceanic area in Troms fylke, northern Norway (69°30′N, 27°20′E), approximately 600–700 m above sea level with dominating Empetrum–Dicranum–Lichen vegetation (Oksanen & Virtanen, 1995). In northern Norway, reindeer are managed by the Sami people and herded for their meat and fur. The reindeer migrate from winter grazing areas in inner Finnmark to summer grazing areas near the coast (Suominen & Olofsson, 2000). During the 1960s, a permanent fence running east–west was constructed in Raisduoddar to prevent reindeer from entering spring and autumn migration ranges during the summer. The fence is made from wire and wooden stakes approximately one meter above the ground and spans several kilometers over the tundra, and the herders let the reindeer pass the fences through certain gates when it is time to migrate. While the side of the fence in the summer range (north) is heavily grazed, the side in the spring and autumn ranges (south) is only lightly grazed due to deep snow during the spring migration and the rapid movement of reindeer toward winter ranges in the autumn (Olofsson et al., 2001). Hereafter, this fence will be referred to as the permanent fence. While the alpine regions in Scandinavia are generally considered to be low‐productive, our study site has calcareous rock, which facilitates the growth of more plant species and thus increases the productivity of the region to intermediate levels compared with conditions generally found in the Scandinavian tundra (Sundqvist et al., 2019).

### 2.2 Study design

**FIGURE 1:**
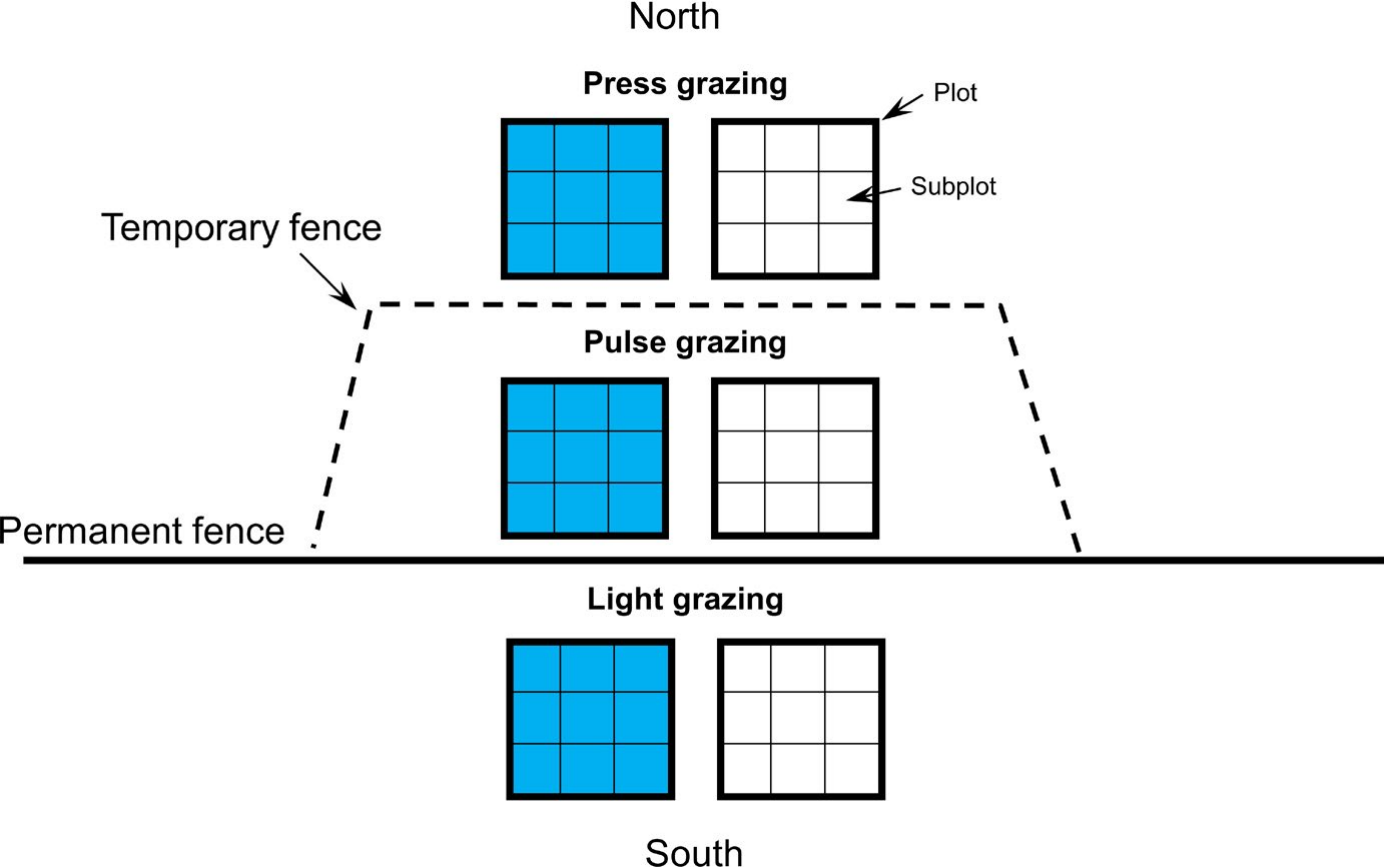
Study design. Plot setup at one temporary fence site. Each site is divided into three grazing regimes by the permanent and temporary fences: light, pulse (i.e., grazed every other year), and press (i.e., grazed every year) grazing. Each grazing regime has two replicate plots: one in a wet area (shown in blue) and one in a dry area (shown in white). Each plot is divided into nine subplots

Our study design consisted of plots set up at five different sites at least 100 m apart from each other along the permanent fence. In 2004, a temporary fence was constructed at each site along the permanent fence, in order to create a pulse treatment extending approximately 6 m into the summer (press)‐grazed side of the fence, before reindeer entered the area in the autumn. Each of the temporary fences is between 85 and 185 meters in length and built in areas that allowed sampling terrain and elevational differences. Each temporary fence is connected to the permanent fence at both ends, which excludes reindeer from entering the area between the temporary and the permanent fences. The temporary fences were taken down every other year to allow for grazing in the area closest to the permanent fence every second year (Figure 1). This was to test the effect of intermittent disturbance on the vegetation. Each site was thus divided into three grazing regimes: The area south of the permanent fence was lightly grazed, the area inside the temporary fence was exposed to acute (i.e., pulse grazing) after more than 40 years of chronic (i.e., press) grazing, and the area outside the temporary fence on the north side continued to experience press grazing. At four of the sites, two replicate plots were placed in each grazing regime (*n* = 6 plots/site). Microtopography created a mixture of drier habitats on ridges and wetter areas in depressions. Thus, for each site one plot was placed in a drier area and other in a wetter area; we refer to this covariate as wet/dry. The last site had four replicate plots placed in each grazing regime (*n* = 12 plots): two in a drier and two in a moist/wet area. Each plot (*n* = 36) was 3 × 3 m (9 m^2^) and was evenly split into nine 1 × 1 m (1 m^2^) subplots (Figure 1). All plots were placed no more than 12 m from the permanent fence, but plots on the lightly grazed side of the fence were placed 3 m away from the fence due to the man‐made disturbance in this area from herders walking next to the fence.

### 2.3 Vegetation data collection

We surveyed the vascular plant vegetation from all the permanent plots in mid‐late July (when most plants were flowering) for each year between 2004 and 2007. All plots were surveyed every year, except for one plot in 2006 and three plots in 2007 due to inadvertent human disturbance, for a total of 140 plot‐level surveys. Surveying was done at the subplot level: The nine subplots of each permanent plot were fully surveyed for species presence/absence data. Species were identified based on *Den nya nordiska floran* (Stenberg & Mossberg, 2003), if, on the rare occasion, an individual plant could not be identified based on what remained it was not recorded. Almost all plants were identified and recorded. Very few plants were unidentifiable and should thus not affect our study. Similarly, few plants could only be identified to the genus level (e.g., *Taraxacum* sp.) and were recorded as such. In only the center subplot of each plot, we surveyed relative species abundance based on plant cover. We used a 50 × 50 cm transparent plexiglass table with 100 randomly distributed 4‐mm‐diameter holes: A pin of the same diameter was lowered through each hole, and the number of contacts the pin had with each species was recorded (see also Olofsson et al., 2001).

### 2.4 Diversity metrics

#### 2.4.1 Species diversity

We measured community richness using: (a) species richness (SR), (b) the Shannon–Wiener diversity index (*H*′) (Shannon & Weaver, 1949), and (c) Smith and Wilson's (1996) index of evenness (*E*_var_). Species presence/absence data were collected for all subplots, so to avoid pseudoreplication, we calculated a single species richness measure for each plot by averaging the species richness of the nine subplots. We chose to average the subplots instead of including the subplots as a nested hierarchical unit in our linear models (see *Statistical Analyses*) because averaging is more robust to minor deviations in the distributions of the data. In addition to calculating the overall species richness of each plot, we calculated the species richness of shrubs (21 species total), herbs (84 species total), and graminoids (42 species total) separately. Our other two measures of community richness required species abundance data, which was only collected for one subplot in each plot, so *H*′ and *E*_var_ measurements at the plot level were taken from a single subplot.

#### 2.4.2 Phylogenetic diversity

##### Phylogeny construction

To study the effect of reindeer grazing on phylogenetic diversity, we constructed a regional vascular plant phylogeny. After reducing all taxonomy assignments to the species level, which involved reassigning subspecies, hybrids, and synonymous species, we identified 147 unique species from our surveys to include in the regional phylogeny. We employed two of the most widely used plant phylogenetic markers, the more slowly evolving chloroplast rbcL gene (Chase et al., 1993) and the faster evolving chloroplast matK gene (Johnson & Soltis, 1994). We searched for sequences for each gene for each species from GenBank (Table A1). If sequence information was not available for one or both of matK and rbcL for a species, we used the closest relative within the same genus with available matK and/or rbcL sequence information. Sequences for the identified species or close relative were available for 138 of 147 species for rbcL and 140 of 147 for matK (Table A1). Two species did not have available sequence information for either gene and did not have a close relative that could be substituted: *Cerastium cerastoides* and *Carex parallela*. *Cerastium cerastoides* was manually inserted at the base of the *Cerastium* clade (Scheen et al., 2004), and *C. parallela* was manually inserted into a clade with *Carex dioica* (Lipnerová et al., 2013).

To ensure the correct reading frame prior to sequence alignment, all sequences were translated to amino acids in ExPASy (Gasteiger et al., 2003). Alignments were completed in MEGA7 (Kumar et al., 2016) using MUSCLE with the nucleotides for the coding regions of plant plastids and the default settings, which are designed to give the most accurate alignments. Noninformative gaps were manually removed from the matK alignments, and excess lengths were trimmed from the ends of both genes. Consistent with other studies (see, e.g., Potter et al., 2007), the Model Selection tool in MEGA7 identified the most flexible substitution model for slowly evolving protein‐coding genes (GTR+G+I) as the best fit to rbcL variation, and the general protein‐coding model (GTR+G) as the best fit to matK variation. These were the substitution models specified in the Bayesian tree inference phase.

**FIGURE A1:**
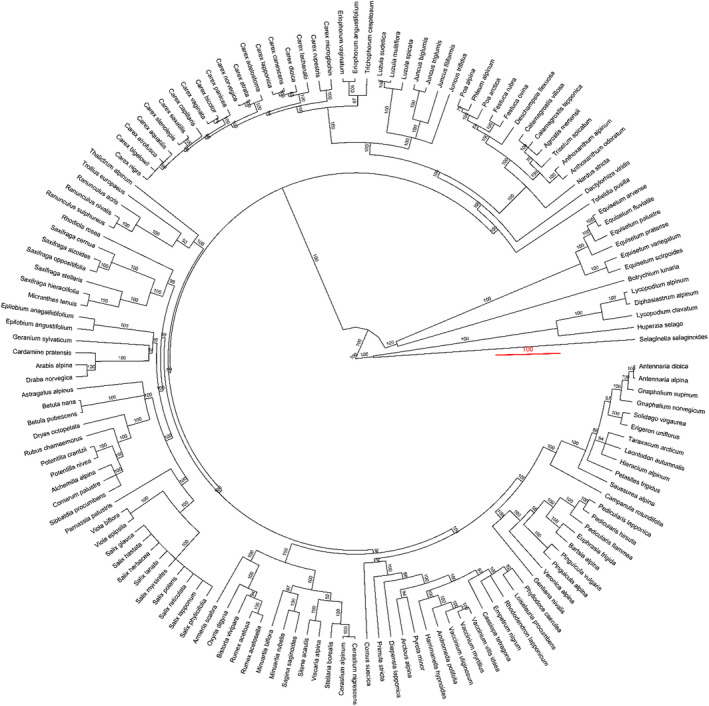
Vascular plant phylogeny. Majority consensus tree, generated with MrBayes (see Section 2), of the 145 vascular plant species sampled in Fennoscandia with available matK and/or rbcL sequences in GenBank. Node values represent posterior probabilities and the red scale bar denotes time in 100 million years

Bayesian inference of trees was performed using MrBayes (Huelsenbeck & Ronquist, 2001) with two partitions: one for matK and one for rbcL. We ran our model for 100,000,000 generations with four chains at a temperature of 0.2 to help explore tree space, with a stop value of 0.01 for convergence. We sampled trees every 1,000 generations, and following standard procedure, we discarded the first 25% of the trees from each chain (the burn‐in period when more poorly fit trees are retained). Our trees were dated by constraining seven nodes (Table 1; Bell et al., 2010). The analysis converged at 10,304,000 generations. For visualization, we generated the majority rule consensus tree by collapsing clades with posterior probabilities less than 50% to polytomies (Figure A1). The phylogeny figure was generated using the “ggtree” (Yu et al., 2017) R package after reading the .tre file from MrBayes using the *read.mrbayes* function in the package “treeio” (Yu, 2019).

**TABLE 1:** Node constraints (in million years) from Bell et al. (2010) input to MrBayes for dating the regional vascular plant phylogeny from Fennoscandia

Node	Minimum	Mean	*SD*	Fixed
Root	407.60	430.82	10.20	NA
Monilophytes	346.70	364.93	8.01	NA
Euphyllophytes	NA	NA	NA	380.00
Angiosperms	113.00	133.27	8.90	NA
Ranunculales	112.00	132.81	9.14	NA
Saxifragales	89.30	101.73	5.46	NA
Caryophyllales	83.50	99.11	6.86	NA

##### Phylogenetic structure

We used mean pairwise distance (MPD) as our measure of phylogenetic structure. MPD is insensitive to species richness, but more sensitive to changes in distantly related taxa than is the mean nearest taxon distance (Webb et al., 2002). To prevent bias resulting from calculations due to distantly related species (e.g., *Lycopodium* species), we used only the angiosperms (133/147 surveyed species) to calculate MPD. We calculated a standardized measure of MPD with the aid of the function *ses.mpd* in the R package “picante” by comparing the observed phylogenetic community structure to a specified null model with a randomized community structure (Kembel et al., 2010; R Core Team, 2019). Using taxa that were identified to the species level, we calculated both (a) a presence/absence‐based measure of MPD (calculated at the subplot level and then averaged within each plot) and (b) an abundance‐based measure (calculated for a single subplot in each plot), by weighting the pairwise distances by the product of the relative abundance of each species in each pair. Both MPD metrics were calculated for every tree in the posterior sample (created by merging the two runs and removing the 25% burn‐in: final *n* = 15,458 trees) and averaged to produce a single measure for each plot. For our null model, we used the *independentswap* algorithm (with 1,000 iterations per run and 999 runs), which randomizes the community data matrix while maintaining species occurrence frequency and sample species richness (Gotelli, 2000). We used the quantile of observed MPD versus the MPD of null communities as our standardized response variable, as this metric is less biased than the more common Net‐Relatedness Index (Vamosi et al., 2014). Our measure describes the rank of the observed phylogenetic dispersion relative to the distribution produced by the null model. A value of 0 corresponds to a community that is more clumped than any of the null communities, a value of 0.5 corresponds to a community that has a median dispersion relative to the null communities, and a value of 1 corresponds to a community that is more dispersed than any of the null communities. We refer to the quantile of observed MPD versus the MPD of null communities as phylogenetic dispersion (from the species presence/absence data) and abundance‐weighted phylogenetic dispersion (from the relative species abundance data).

### 2.5 Statistical analysis

#### 2.5.1 Species and phylogenetic diversity

We analyzed the effect of grazing intensity on SR (overall and for the shrubs, herbs, and graminoids), *H*′, *E*_var_, phylogenetic dispersion, and abundance‐weighted phylogenetic dispersion using linear mixed‐effects models with grazing, year, a grazing:year interaction, and wet/dry as fixed effects. In each model, we also included site as a random effect and nested an additional variable that reflects the combination of site and grazing regime within site to account for our hierarchical study design. First, we tested the grazing:year interaction using a likelihood ratio test. If the interaction was not significant (*p* > .05), then we removed it from the model and tested the remaining fixed effects. All the remaining fixed effects were retained in the model regardless of their significance. If a significant effect of grazing or year was found (*p* < .05), then a post hoc analysis was performed with Tukey's pairwise comparisons. To control for the possibility that changes in phylogenetic dispersion may result from the transition of communities from dwarf shrubs to graminoids, we included the proportion of graminoid species (for phylogenetic dispersion) and the graminoid relative abundance (for abundance‐weighted phylogenetic dispersion) as covariates in their respective models. This allowed us to test for changes in phylogenetic dispersion that were independent of the transition in community composition from dwarf shrubs to graminoids. The means estimated from the linear models, that is, the least squared means (lsmeans), and the 95% confidence intervals were estimated using the “lsmeans” (Lenth, 2016) R package. The normality and homogeneity of the residuals were graphed for each model to check that model assumptions were met.

#### 2.5.2 Community structure

To compare the community structure of plots, we used the Bray–Curtis dissimilarity index, calculated using *vegdist* from the R package “vegan” (Oksanen et al., 2020), as a measurement of the distance between plant communities based on our relative species abundance data. To partition variance within the distance matrix, we used a nonparametric permutational multivariate analysis of variance (PERMANOVA), as implemented in the vegan function *adonis*. Significance values and pseudo‐F‐statistics were obtained from permutations (*n* = 1,000) restricted within each site due to our nested study design. Grazing, year, a grazing:year interaction, and wet/dry were included as covariates. When significant values (*p* < .05) were obtained, we performed a post hoc analysis with Bonferroni corrections to correct for multiple comparisons in the PERMANOVA.

To visualize and corroborate the results of the PERMANOVA, we used a nonmetric multidimensional scaling (NMDS) from the function *metaMDS* in vegan. NMDS is an ordination technique that represents highly dimensional data by maximizing the correlation of ranked distances between the original highly dimensional data and a two‐dimensional representation (Faith et al., 1987; Legendre & Legendre, 2012; Minchin, 1987). A stress score is calculated as a measure of how accurately the two‐dimensional ordination represents the distances in the original data; stress scores <0.2 are generally considered acceptable (Clarke, 1993). Communities grouped closely together in the ordination space are interpreted as being more similar than those placed farther away. Confidence ellipses were drawn with the vegan function *ordiellipse* using the standard deviations and a confidence limit of 0.75. All analyses were performed in R version 3.6.0 (R Core Team, 2019).

## 3 RESULTS

### 3.1 Biodiversity across grazing regimes

All three of our plant richness metrics, species richness (mean ± *SD* (range): 20.75 ± 7.26 (6.22–41.11) species), the Shannon–Wiener diversity index, that is, *H*′ (1.79 ± 0.56 (0.33–2.88)), and the index of evenness, that is, *E*_var_ (0.41 ± 0.11 (0.16–0.98)), varied markedly among plant communities. However, analyses using linear mixed‐effect models revealed that grazing intensity did not explain a significant amount of this variation (Table 2). Species richness was greater in 2004 (lsmean (95% CI): 24.7 (21.2–28.2) species) than in 2005 (20.4 (16.9–23.8) species; *p* = .04), 2006 (19.2 (15.7–22.8) species; *p* = .005), and 2007 (18.8 (15.2–22.4) species; *p* = .002). Similarly, *H*′ was greater in 2004 (2.13 (1.89–2.37)) than in 2006 (1.59 (1.35–1.84); *p* < .0001) and 2007 (1.49 (1.24–1.74); *p* < .0001), and plant communities from 2005 (1.93 (1.69–2.17)) were more diverse than plant communities from 2006 (*p* = .02) and 2007 (*p* = .001). *H*′ was also greater in dry (1.89 (1.70–2.08)) than in wet (1.68 (1.49–1.88)) plant communities.

**TABLE 2:** Results from linear mixed‐effects models testing the effect of grazing intensity on three biodiversity metrics for plant communities in Fennoscandia: species richness, the Shannon–Wiener diversity index (*H*′), and Smith and Wilson’s (1996) index of evenness (*E*_var_)

Biodiversity metric	Model term	*F*‐value	*df*	*p*
Species richness	Grazing	0.35	2,10	.71
	**Year**	**5.58**	**3,118**	**.001**
	Grazing:Year	0.13	6,112	.99
	Wet/dry	0.001	1,118	.97
*H*′	Grazing	2.04	2,10	.18
	**Year**	**13.34**	**3,118**	**< .0001**
	Grazing:Year	0.32	6,112	.92
	**Wet/dry**	**5.51**	**1,118**	**.02**
*E*_var_	Grazing	0.78	2,10	.48
	Year	0.36	3,118	.78
	Grazing:Year	0.58	6,112	.74
	Wet/dry	0.68	1,118	.41

Note

Model terms that are significant (*p* < .05) are bolded, *df* = degrees of freedom.

### 3.2 Community structure across grazing regimes

**FIGURE 2:**
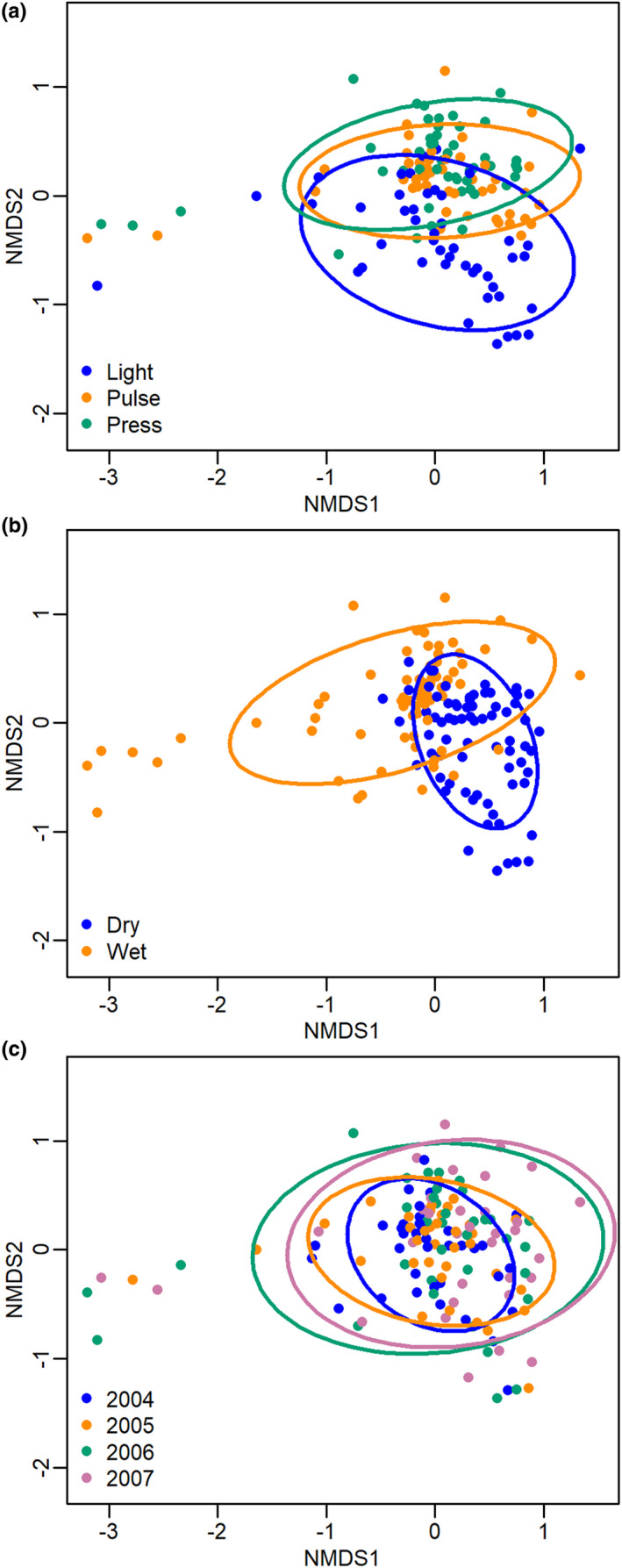
Plant community structure is altered by pulse and press reindeer grazing and in wet versus dry plots. Nonmetric multidimensional scaling (NMDS) of a Bray–Curtis distance matrix describing vascular plant communities in Fennoscandia. Each point symbolizes a plant community from an individual plot (*n* = 140), and colors display the characteristics of each community: (a) communities with light, pulse, or press reindeer grazing, (b) communities in wet or dry areas, and (c) the year the community was surveyed in. The ellipses represent confidence ellipses for each group. The stress value is 0.18

Grazing intensity explained a significant amount of the variation in the overall vascular plant community structure in the PERMANOVA (Table 3; Figure 2a). Post hoc analysis revealed that the effect of grazing intensity on community structure was significant between plant communities with light and pulse grazing (Bonferroni‐adjusted *p* = .003) and between plant communities with light and press grazing (Bonferroni‐adjusted *p* = .003), but not between communities with press and pulse grazing (Bonferroni‐adjusted *p* = .27; Figure 2a). Wet/dry and year also explained a significant amount of variation in the vascular plant community structure (Table 3; Figure 2b,c). The effect of year on community structure was only significant between plant communities in 2004 and 2007 (Bonferroni‐adjusted *p* = .006; Figure 2c).

**TABLE 3:** Results from the permutational multivariate analysis (PERMANOVA) of the Bray–Curtis dissimilarities for vascular plant community structure, constrained by site, in relation to wet/dry, grazing, year, and the grazing:year interaction, *df* = degrees of freedom; SS = sum of squares; MS = mean sum of squares; Pseudo‐*F* = *F*‐value from permutations

Variable	*df*	SS	MS	Pseudo‐*F*	*p*
Grazing	2	3.64	1.82	5.58	.001
Year	3	1.67	0.56	1.71	.002
Grazing:Year	6	0.78	0.13	0.40	1
Wet/dry	1	3.15	3.15	9.66	.001
Residual	127	41.45	0.33		
Total	139	50.69			

Note

*p*‐values are based on 1,000 permutations.

**FIGURE 3:**
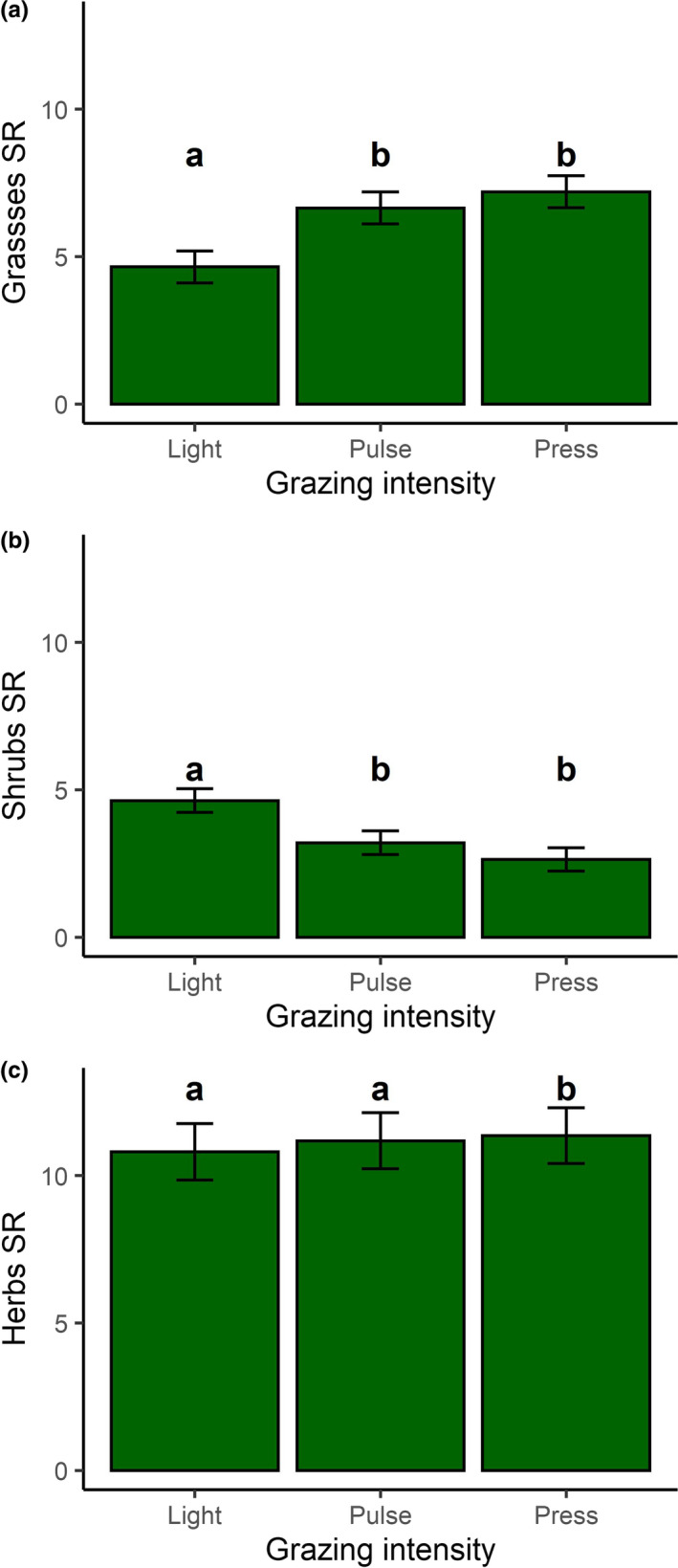
Shrub species are replaced by grass species in communities with pulse and press grazing. The effect of reindeer grazing on a) graminoid species richness, b) shrubs species richness, and c) herbs species richness in Fennoscandia. The values shown are the lsmeans ±SE, derived from linear mixed‐effects models. Different numbers indicate statistical significance between groups at the *p* < .05 level, tested using Tukey's test for multiple comparisons

In addition to the variation in each of the above metrics of overall plant richness, there was also substantial variation among plant communities in the mean species richness of shrubs (mean ± *SD* (range): 3.55 ± 1.84 (0.11–9.00) species), herbs (11.05 ± 5.22 (1.56–22.56) species), and graminoids (6.15 ± 2.63 (1.44–12.56) species). Grazing intensity explained a significant amount of the variation in graminoid and shrub species richness (Figure 3 and Table 4): Plant communities with light grazing (lsmean (95% CI): 4.65 (3.25–6.05) species) had fewer graminoid species than plant communities with pulse (6.65 (5.36–8.04) species; *p* = .005) and press (7.20 (5.80–8.59) species; *p* = .0008) grazing. In contrast, plant communities with light grazing (4.63 (3.60–5.66 species) had more shrub species than communities with pulse (3.20 (2.18–4.23) species; *p* = .02) and press (2.64 (1.61–3.66) species; *p* = .002) grazing (Figure 3). There was no variation in the species richness of herbs with grazing intensity (Figure 3 and Table 4). Graminoid species richness varied with year (Table 4) as communities from 2004 (7.84 (6.44–9.22 species) had more species of graminoids than communities from 2005 (6.00 (4.61–7.39) species; *p* = .0003), 2006 (5.75 (4.35–7.15); *p* < .0001), and 2007 (5.08 (3.67–6.50) species; *p* < .0001). Finally, shrubs species richness varied with wet/dry (Table 4) as dry communities (4.23 (3.38–5.09) species) had more shrub species than wet communities (2.75 (1.89–3.61) species).

**TABLE 4:** Results from linear mixed‐effects models testing the effect of grazing intensity on the species richness of graminoids, shrubs, and herbs for plant communities in Fennoscandia

Plant group	Model term	*F*‐value	*df*	*p*
Graminoids	**Grazing**	**16.05**	**2,10**	**.0008**
	**Year**	**13.63**	**3,118**	**<.0001**
	Grazing:Year	0.33	6,112	.92
	Wet/dry	0.05	1,118	.81
Shrubs	**Grazing**	**11.91**	**2,10**	**.002**
	Year	2.12	3,118	.10
	Grazing:Year	0.11	6,112	.99
	**Wet/dry**	**52.24**	**1,118**	**<.0001**
Herbs	Grazing	0.15	2,10	.86
	Year	2.30	3,118	.08
	Grazing:Year	0.07	6,112	.99
	Wet/dry	2.70	1,118	.10

Note

Model terms that are significant (*p* < .05) are bolded, *df* = degrees of freedom.

### 3.3 Phylogenetic structure across grazing regimes

**FIGURE 4:**
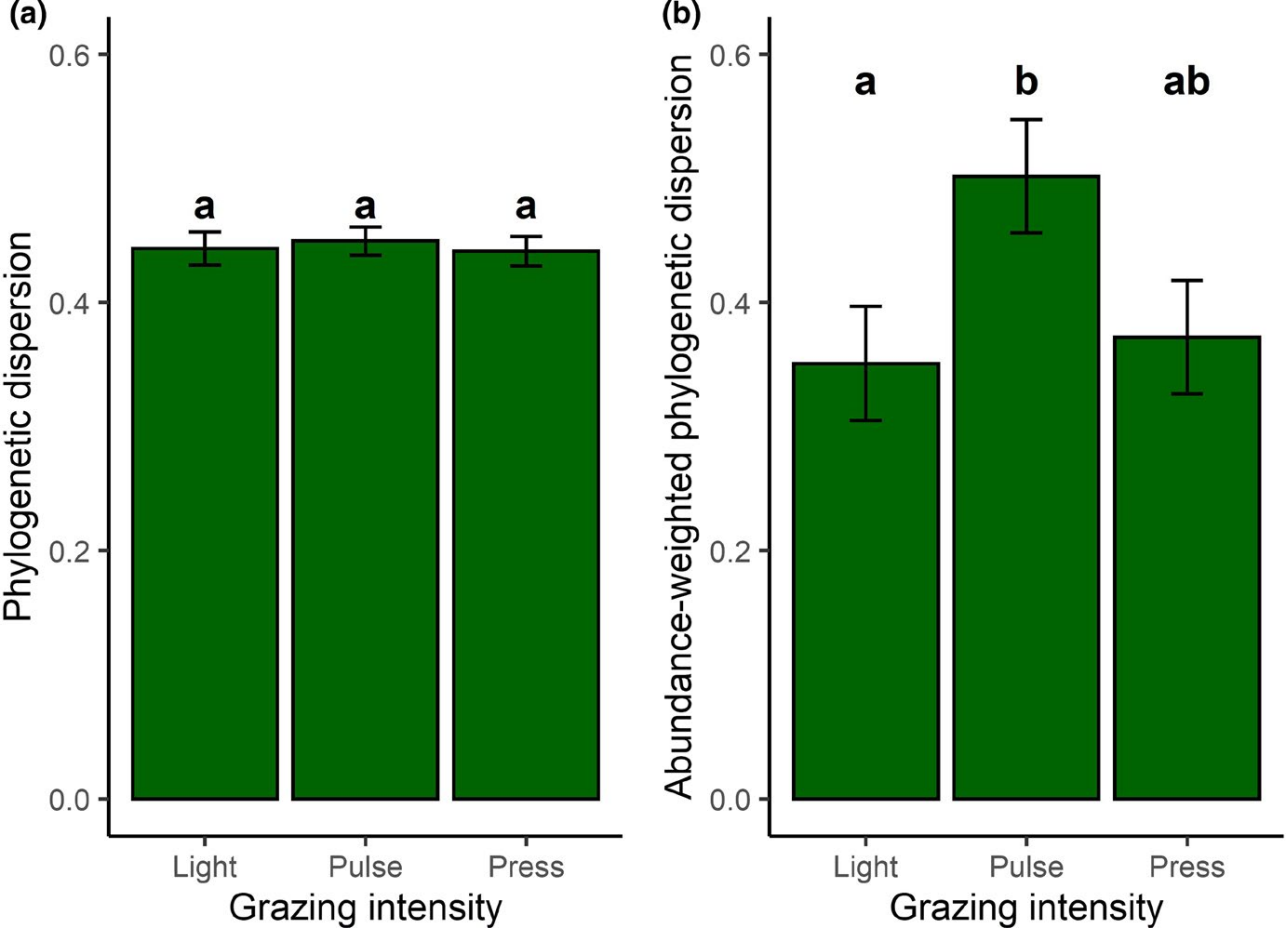
Phylogenetic dispersion of plant communities increases in communities with pulse but not press grazing. The effect of reindeer grazing on a) phylogenetic dispersion (calculated from species presence/absence data) and b) abundance‐weighted phylogenetic dispersion (calculated from relative species abundance data) of vascular plant communities in Fennoscandia. Values of phylogenetic dispersion close to 0 represent phylogenetically clumped communities, while values close to 1 represent phylogenetically dispersed communities. The values shown are the lsmeans ± *SE*, derived from linear mixed‐effects models. Different numbers indicate statistical significance between groups at the *p* < .05 level, tested using Tukey's test for multiple comparisons

Abundance‐weighted phylogenetic dispersion (mean ± *SD* (range): 0.41 ± 0.26 (0.03–0.95)), but not unweighted phylogenetic dispersion (mean ± *SD* (range): 0.44 ± 0.08 (0.24–0.60)), varied with grazing intensity (Figure 4 and Table 5). Using the abundance‐weighted metric, plant communities with pulse grazing (lsmean (95% CI): 0.52 (0.49–0.63)) were more phylogenetically dispersed than communities with light grazing (0.29 (0.16 = 0.41); *p* = .006), while there was no significant difference between communities with light and press grazing (0.43 (0.30–0.55); *p* = .11) or between pulse and press grazing (*p* = .27). Additionally, wet plant communities were more clumped (0.38 (0.27–0.49)) than dry communities (0.44 (0.34–0.55)). Finally, using the unweighted metric, communities from 2005 (0.46 (0.43–0.49)) were more phylogenetically dispersed than communities from 2004 (0.41 (0.38–0.45); *p* = .03).

**TABLE 5:** Results from linear mixed‐effects models testing the effect of grazing intensity on the phylogenetic dispersion (calculated from species presence/absence data) and abundance‐weighted phylogenetic dispersion (calculated from relative species abundance data) of plant communities in Fennoscandia

Metric	Model term	*F*‐value	*df*	*p*
Phylogenetic dispersion	Grazing	0.16	2,10	.86
	**Year**	**3.06**	**3,117**	**.03**
	Grazing:Year	1.53	6,111	.17
	Wet/dry	0.28	1,117	.60
	Graminoids	0.36	1,117	.55
Abundance‐weighted phylogenetic dispersion	Grazing	8.21	2,10	.008
	Year	0.85	3,117	.47
	Grazing:Year	0.42	6,111	.87
	**Wet/dry**	**5.22**	**1,117**	**.02**
	Graminoids	15.71	1,117	.0001

Note

The Graminoids terms refer to the proportion of graminoid species (for phylogenetic dispersion) and the graminoid relative abundance (for abundance‐weighted phylogenetic dispersion). Model terms that are significant (*p* < .05) are bolded, *df* = degrees of freedom.

## 4 DISCUSSION

We report for the first time that the intensity of reindeer grazing has effects on the phylogenetic community structure of grazed plant communities that are not captured by standard diversity indexes. Plant communities grazed every other year (i.e., pulse grazing), but not communities grazed every year (i.e., press grazing) were more phylogenetically dispersed than lightly grazed communities, while there was no effect of grazing on species richness, the Shannon‐Wiener index (*H*′), and community evenness (*E*_var_). This does not support our predictions that grazing would act as a biotic filter, resulting in communities that are more phylogenetically clustered, but rather suggests that pulses of grazing allow more phylogenetically diverse communities.

We observed no effect of grazing on species richness, the Shannon–Wiener index (*H*′), and community evenness (*E*_var_). Since the productivity of our study site is intermediate compared with the sites included in a multisite study over the Fennoscandian tundra (Sundqvist et al., 2019), the neutral effect of reindeer grazing on species diversity reported here is in agreement with a previous study (Sundqvist et al., 2019), as well as general reviews of the effects of herbivores on plant diversity that predict that increased grazing will have no effect or a negative effect on biodiversity in the nutrient‐poor tundra (Bernes et al., 2015; Proulx & Mazumder, 1998).

While we did not observe a change in the overall richness and evenness of vascular plants across grazing regimes, we did detect an effect of grazing on the species composition of communities. Communities with pulse and press grazing both differed in community structure from lightly grazed communities (Figure 2a) as reindeer grazing induced a transition from shrub‐dominated to graminoid‐dominated communities (Figure 3). These results are fully consistent with previous studies in Fennoscandia that report the replacement of dwarf shrubs by graminoids in heavily grazed areas (Olofsson et al., 2001, 2004). Similar effects of grazing on vegetation composition have also been observed in other systems (Begley‐Miller et al., 2014; Clarke et al., 1995; Ferreira et al., 2020; McKendrick et al., 1980; Rooney, 2009; van der Wal, 2006; van det Wal et al., 2014). For example, grazing by sheep in Great Britain has resulted in the replacement of heather moorlands with graminoids (Clarke et al., 1995) and grazing by mammalian herbivores in northern Alaska also resulted in the replacement of tundra heaths by graminoids (McKendrick et al., 1980). Potential reasons for this shift in vegetation composition include increased soil nutrient concentrations that favor graminoids (McKendrick et al., 1980) and an increased ability of graminoids to outcompete shrubs and ferns in heavily grazed environments due to their short stature, high shoot densities, and capacity for compensatory growth (Coughenour, 1985). This combination of traits allows graminoids to be the first plants to colonize following disturbances (Chapin & Shaver, 1981). The shift from shrubs to graminoids was also observed in our communities with several years of pulse grazing (Figure 2a; Figure 3). This means that pulses of grazing over a period of several years did not change the community that was established by decades of chronic grazing before the temporary fences were established.

Our novel analysis of the effect of reindeer grazing on the phylogenetic structure of vascular plant communities revealed that communities with pulse, but not press grazing seem more phylogenetically dispersed than lightly grazed communities (Figure 4). This is in contrast to our prediction that, given antiherbivore traits are evolutionarily conserved (Loiola et al., 2012; Yessoufou et al., 2013, but see Kursar et al., 2009) and reindeer are generalist herbivores (Baskin & Danell, 2003), press reindeer grazing would result in communities that are more phylogenetically clustered compared with lightly grazed communities (Cavender‐Bares et al., 2009). Other studies examining the effect of grazing on phylogenetic structure have found conflicting results. Grazing by white‐tailed deer (*Odocoileus virginianus*) resulted in phylogenetic clumping (Begley‐Miller et al., 2014), while grazing by large herbivores in the African savanna resulted in changes in phylogenetic community structure dependent on the initial community structure: Communities that were initially clumped became more dispersed, while communities that were initially dispersed became more clumped (Yessoufou et al., 2013). In contrast, grazing by livestock had no effect on the phylogenetic dispersion of plant communities in Chile (Salgado‐Luarte et al., 2019). One potential explanation for our result is that antiherbivore defense traits are actually evolutionarily convergent in our study region, a situation where grazing by a generalist herbivore would result in phylogenetic overdispersion (Cavender‐Bares et al., 2009). However, it should be noted that the predicted effect of grazing on the phylogenetic structure of plant communities by Cavender‐Bares et al. (2009) is based only on the direct effects of herbivory (i.e., the physical removal of species) and does not account for the indirect effects on, for example, productivity, nutrient availability, trampling disturbance, and competitive interactions between species. For example, if herbivory increases soil nutrient availability and decreases competition between plant species (both indirect effects of grazing), this might lead to phylogenetic dispersion, given that both competition between distantly related taxa (Mayfield & Levine, 2010) and limited nutrient availability (Hurteau et al., 2016) may drive phylogenetic clumping. In this case, the indirect effects of grazing by herbivores on phylogenetic structure may act in the opposite direction of the direct effects of grazing, and the resulting phylogenetic structure may depend on the relative strength of the direct and indirect effects. Given that we found that pulse, but not press, grazing results in increased phylogenetic dispersion, it is possible that in our study system the indirect effects of reindeer grazing are more important than the direct effects in driving community assembly for plant communities exposed to acute periods of grazing after decades of chronic grazing. This would mean that the direct effects of reindeer grazing are stronger if grazing happens every year, but not if communities are exposed to acute grazing periods. This could transpire if acute grazing periods briefly promote phylogenetic clustering, but are followed by periods without grazing where the persisting indirect effects of grazing (on, e.g., soil nutrient availability) promote phylogenetic dispersion.

To be able to manipulate reindeer grazing regimes and achieve the pulse and press treatments, we collaborated with reindeer herders and so were restricted in our experimental design to take advantage of existing infrastructure. While the short‐term fences creating the pulse treatment can be regarded as a randomized experiment, the long‐term fence should be characterized as a quasi‐experimental design, which may pose a challenge in interpreting results (Krebs, 2014). Here, we consider the advantage of realistic large‐scale treatments outweighs potential drawbacks with the experimental design, and that the distance between sites along the long‐term fence (>100 m) allows us to consider these units as statistically independent replicates of local plant species composition.

The results of this study may be relevant to management decisions involving reindeer in Fennoscandia since the patterns imply that we should consider not only grazing intensity but also grazing regime (press or pulse), and not only traditional diversity measures but also phylogenetic diversity. In addition, the effects of pulse grazing were apparent after four years only, indicating that management decisions can have effects on the structure and function of these arctic ecosystems in the short term. While we did not observe any effects of reindeer grazing on plant species richness and diversity in our study area, we do observe a significant effect of grazing on vegetation composition and phylogenetic structure. More specifically, several years of pulse grazing (following over 40 years of chronic grazing) increased the phylogenetic dispersion of vascular plant communities compared to communities with almost no grazing and communities with continued press grazing. Changes to the phylogenetic structure of communities may have important consequences for community function if phylogenetic diversity captures genetic and functional diversity related to ecosystem productivity and, for example, resilience (see, e.g., Cadotte et al., 2009; Flynn et al., 2011). Given the numerous studies that have analyzed the impact of reindeer herbivory on vegetation composition, there is a potential to re‐analyze existing datasets using the framework of phylogenetic community ecology as done here. Additionally, future studies considering variation in productivity and grazing intensity will test whether general relationships between grazing and phylogenetic diversity exist in the same way they do for common diversity measures, or whether the responses depend on other factors such as the evolutionary history of plants and herbivores. Direct measurements of ecosystem functions in such communities are also needed to help understand the mechanisms driving plant community assembly in support of future management decisions.

## CONFLICT OF INTEREST

The authors declare no conflicts of interest.

## AUTHOR CONTRIBUTIONS

**Kate Gibson:** Formal analysis (lead); Visualization (lead); Writing‐original draft (lead). **Johan Olofsson:** Data curation (supporting); Writing‐review & editing (supporting). **Arne Ø. Mooers:** Supervision (supporting); Writing‐review & editing (supporting). **Melanie J. Monroe:** Conceptualization (lead); Data curation (lead); Funding acquisition (lead); Supervision (lead); Writing‐review & editing (lead).

## Data Availability

Data have been uploaded to Dryad (https://doi.org/10.5061/dryad.dbrv15f24).

## APPENDIX A

**TABLE A1:** List of species included in our regional vascular plant phylogeny inferred from matK and rbcL sequences obtained from GenBank

Family	Species	matK accession	rbcL accession
Asteraceae	*Antennaria alpina*	KC474013	KC481938
Asteraceae	*Antennaria dioica*	HM445620.MATK	HE574602
Asteraceae	*Erigeron uniflorus*	KC474720	KC482770
Asteraceae	*Gnaphalium norvegicum*	JN895615 (*Gnaphalium uliginosum*)	KM360808.1 (*Gnaphalium uliginosum*)
Asteraceae	*Gnaphalium supinum*	HM445621.MATK	KF997337
Asteraceae	*Hieracium alpinum*	AJ633201	JQ933362 (*Hieracium umbellatum*)
Asteraceae	*Leontodon autumnalis*	AJ633220	HE574613
Asteraceae	*Petasites frigidus*	JN966416	JN965722
Asteraceae	*Saussurea alpine*	KC590037	KC589890.RBCL
Asteraceae	*Solidago virgaurea*	JN895829	HE574593
Asteraceae	*Taraxacum arcticum*	FJ395377 (*Taraxacum officinale*)	KT960668
Betulaceae	*Betula nana*	AY372021.MATK	KC482102
Betulaceae	*Betula pubescens*	AY372025.MATK	KX162898
Brassicaceae	*Arabis alpine*	AF144328	HF934132.RBCL
Brassicaceae	*Cardamine pratensis*	HM850749	KM360692
Brassicaceae	*Draba norvegica*	KJ840908	KC482623
Campanulaceae	*Campanula rotundifolia*	KT176623	KT178143
Caryophyllaceae	*Cerastium alpinum*	JN894309	KC482406
Caryophyllaceae	*Cerastium cerastoides*	NA	NA
Caryophyllaceae	*Cerastium nigrescens*	NA	KF997275
Caryophyllaceae	*Minuartia biflora*	KC475007	KC483175
Caryophyllaceae	*Minuartia rubella*	KC475016	KC483193
Caryophyllaceae	*Sagina saginoides*	KF737610.MATK	KF997390
Caryophyllaceae	*Silene acaulis*	EF547235.MATK	KC484097
Caryophyllaceae	*Stellaria borealis*	JN589285.1	M62570.1 (*Stellaria media*)
Caryophyllaceae	*Viscaria alpine*	FJ589569	KC484324
Celastraceae	*Parnassia palustris*	AY935910	AY935731
Cornaceae	*Cornus suecica*	DQ341334	AF421085
Crassulaceae	*Rhodiola rosea*	JN895351	KM360979
Cyperaceae	*Carex adelostoma*	LK021877.MATK	NA
Cyperaceae	*Carex aquatilis*	KP273666	FJ548247
Cyperaceae	*Carex atrata*	LK021879.MATK	JX644605
Cyperaceae	*Carex atrofusca*	KT021439.MATK	FJ548249
Cyperaceae	*Carex bicolor*	LK021889.MATK	JN965343
Cyperaceae	*Carex bigelowii*	LK021890.MATK	FJ548252
Cyperaceae	*Carex canescens*	KP980061	GQ469845
Cyperaceae	*Carex capillaris*	JN966190	FJ548253
Cyperaceae	*Carex dioica*	JN895942	JN890670
Cyperaceae	*Carex lachenalii*	KP979990	FJ548261
Cyperaceae	*Carex lapponica*	KP979993	NA
Cyperaceae	*Carex microglochin*	KP273698	GQ469844
Cyperaceae	*Carex nigra*	FN668463	GQ469838
Cyperaceae	*Carex norvegica*	LK021919.MATK	KC482327
Cyperaceae	*Carex panicea*	LK021925.MATK	KM360694
Cyperaceae	*Carex rupestris*	KJ513591	FJ548278
Cyperaceae	*Carex parallela*	NA	NA
Cyperaceae	*Carex saxatilis*	HG915878.MATK	FJ548280
Cyperaceae	*Carex stenolepis*	HG915881.MATK	NA
Cyperaceae	*Carex vaginata*	JN966212	JN965414
Cyperaceae	*Eriophorum angustifolium*	KJ513597	JX644681
Cyperaceae	*Eriophorum vaginatum*	JN895096.1	AB369971.1
Cyperaceae	*Trichophorum cespitosum*	KJ513657	AB369974
Diapensiaceae	*Diapensia lapponica*	KU195972.MATK	DAECPRBCLA
Equisetaceae	*Equisetum arvense*	GU191334.MATK	EARCPRBCL.RBCL
Equisetaceae	*Equisetum fluviatile*	NA	AY226142
Equisetaceae	*Equisetum palustre*	AM883541	AY226138
Equisetaceae	*Equisetum pratense*	AM883553.1 (*Equisetum sylvaticum*)	AY226137
Equisetaceae	*Equisetum scirpoides*	AM883552	AY226133
Equisetaceae	*Equisetum variegatum*	AM883554	AY226134
Ericaceae	*Andromeda polifolia*	AF124569	AF124572
Ericaceae	*Arctous alpine*	JN966111	KM360646
Ericaceae	*Harrimanella hypnoides*	HHU61315	HHU82766
Ericaceae	*Cassiope tetragona*	KC474415	KC482389
Ericaceae	*Empetrum nigrum*	AF519558	AF421091
Ericaceae	*Loiseleuria procumbens*	LPU61352	LPU49288
Ericaceae	*Phyllodoce caerulea*	PCU61318	AB842057
Ericaceae	*Pyrola minor*	JN894990	KM360950
Ericaceae	*Rhododendron lapponicum*	JN966495	JN965809
Ericaceae	*Vaccinium myrtillus*	AF382810	KM361028
Ericaceae	*Vaccinium uliginosum*	AF419717	AF421107
Ericaceae	*Vaccinium vitis‐idaea*	AF382819	AF419837
Fabaceae	*Astragalus alpinus*	AY920438	KM360658
Gentianaceae	*Gentiana nivalis*	EF552121	DQ660644.1 (*Gentiana verna*)
Geraniaceae	*Geranium sylvaticum*	EU922315.1 (*Geranium palmatum*)	KF997286
Juncaceae	*Juncus biglumis*	NA	KC483019
Juncaceae	*Juncus filiformis*	JN895484	JN892294
Juncaceae	*Juncus trifidus*	AY973526	AY216618
Juncaceae	*Juncus triglumis*	JN894676	AY216605
Juncaceae	*Luzula multiflora*	JN895525	AJ419945.RBCL
Juncaceae	*Luzula spicata*	KJ840936	AY216645
Juncaceae	*Luzula sudetica*	AY973519.MATK	AY216647
Lentibulariaceae	*Pinguicula alpine*	AF531783.MATK	AF482524.1 (*Pinguicula gracilis*)
Lentibulariaceae	*Pinguicula vulgaris*	AF531806.MATK	KM360935
Lycopodiaceae	*Diphasiastrum alpinum*	EU749489.1 (*Diphasiastrum digiatum*)	KC482537.1
Lycopodiaceae	*Lycopodium alpinum*	NA	AJ133250
Lycopodiaceae	*Huperzia selago*	DQ465962	DQ464227
Lycopodiaceae	*Lycopodium clavatum*	KT821305	Y07936
Onagraceae	*Epilobium anagallidifolium*	KP682439.MATK	KF997410
Onagraceae	*Epilobium angustifolium*	KP682441.MATK	KM360765
Ophioglossaceae	*Botrychium lunaria*	KP757848.1	DQ849146
Orchidaceae	*Dactylorhiza viridis*	KJ452797.MATK	KJ451495.RBCL
Orobanchaceae	*Bartsia alpine*	AY849600.MATK	KU235112
Orobanchaceae	*Euphrasia frigida*	KC474788	KT960525
Orobanchaceae	*Pedicularis flammea*	HG423940.MATK	JN965711
Orobanchaceae	*Pedicularis hirsute*	KC475208	KT022766
Orobanchaceae	*Pedicularis lapponica*	HG423970.MATK	JN965719
Plantaginaceae	*Veronica alpine*	HQ593489.1 (*Veronica officinalis*)	KF997333
Plumbaginaceae	*Armeria scabra*	KC474091	KC482023
Poaceae	*Agrostis mertensii*	DQ146805.MATK	KC481888
Poaceae	*Anthoxanthum odoratum*	AM234541.MATK	KM974732.RBCL
Poaceae	*Anthoxanthum alpinum*	DQ786884.1 (*Anthoxanthum odoratum*)	KF522673
Poaceae	*Calamagrostis lapponica*	KC474233	KC482158
Poaceae	*Calamagrostis villosa*	KF713112.1 (*Calamagrostis epigeios*)	KJ204308.1 (*Calamagrostis epigeios*)
Poaceae	*Deschampsia flexuosa*	AM234545.MATK	KJ841123
Poaceae	*Festuca ovina*	JX871940.MATK	JX871940.RBCL
Poaceae	*Festuca rubra*	DQ786911	AJ746261
Poaceae	*Nardus stricta*	AF164394	HM850196
Poaceae	*Phleum alpinum*	KM974747.MATK	KM974747.RBCL
Poaceae	*Poa alpine*	DQ786933	JN965736
Poaceae	*Poa arctica*	JN966435	JN965740
Poaceae	*Trisetum spicatum*	KC476082	KJ841637
Polygonaceae	*Oxyria digyna*	EU840459.MATK	KM360910
Polygonaceae	*Bistorta vivipara*	EU840456.MATK	FM883608
Polygonaceae	*Rumex acetosa*	KX095187	KC817303.RBCL
Polygonaceae	*Rumex acetosella*	JN893966	HM850316
Primulaceae	*Primula stricta*	KC475524	AF394975
Ranunculaceae	*Ranunculus acris*	AY954199.MATK	AY395557
Ranunculaceae	*Ranunculus nivalis*	AY954123.MATK	KC483849
Ranunculaceae	*Ranunculus sulphurous*	FM242752.MATK	KC483867
Ranunculaceae	*Thalictrum alpinum*	JN895143	JX258331
Ranunculaceae	*Trollius europaeus*	AY515236	HE574595
Rosaceae	*Alchemilla alpine*	KF997340	KF997346
Rosaceae	*Comarum palustre*	JN896178	KF724289
Rosaceae	*Dryas octopetala*	JF317424	JF317483
Rosaceae	*Potentilla crantzii*	JN896011	JN893502
Rosaceae	*Potentilla nivea*	JN966465	GQ436607
Rosaceae	*Rubus chamaemorus*	AY366358.MATK	JN965826
Rosaceae	*Sibbaldia procumbens*	KC475875	KF997360
Salicaceae	*Salix glauca*	KM002138	KM003004
Salicaceae	*Salix hastate*	KX016203	AB012786
Salicaceae	*Salix herbacea*	EU790670.MATK	JN965906
Salicaceae	*Salix lanata*	JN966591	HE612049
Salicaceae	*Salix lapponum*	NA	GU373339
Salicaceae	*Salix myrsinites*	GU373368	KF997500
Salicaceae	*Salix phylicifolia*	NA	GU373349
Salicaceae	*Salix polaris*	KM002291	FJ788569
Salicaceae	*Salix reticulate*	EU790672.MATK	AJ235793
Saxifragaceae	*Saxifraga aizoides*	KC475819	KM360971
Saxifragaceae	*Saxifraga cernua*	SAXCPMATKA	SCU06215
Saxifragaceae	*Saxifraga hieraciifolia*	AF115485	KC483155
Saxifragaceae	*Saxifraga oppositifolia*	SAXCPMATKD	SOU06217
Saxifragaceae	*Saxifraga stellaris*	AF115493	KC749991.RBCL
Saxifragaceae	*Micranthes tenuis*	KC474994	KC483165
Selaginellaceae	*Selaginella selaginoides*	KR028119.1 (*Selaginella uncinata*)	AF419048
Tofieldiaceae	*Tofieldia pusilla*	AB541043.MATK	AJ286562.RBCL
Violaceae	*Viola biflora*	DQ842607.1	HM850467.1
Violaceae	*Viola epipsila*	JN966733.1 (*Viola renifolia*)	JN966062.1 (*Viola renifolia*)

Note

GenBank accession IDs with a different species name in brackets indicate that the sequence is from a different, but closely related, species. NA values indicate the absence of an available GenBank sequence.
